# Whole Genome Amplification and Reduced-Representation Genome Sequencing of *Schistosoma japonicum* Miracidia

**DOI:** 10.1371/journal.pntd.0005292

**Published:** 2017-01-20

**Authors:** Jonathan A. Shortt, Daren C. Card, Drew R. Schield, Yang Liu, Bo Zhong, Todd A. Castoe, Elizabeth J. Carlton, David D. Pollock

**Affiliations:** 1 Department of Biochemistry & Molecular Genetics, University of Colorado School of Medicine, Aurora, CO, United States of America; 2 Department of Biology, University of Texas Arlington, Arlington, TX, United States of America; 3 Institute of Parasitic Disease, Sichuan Center for Disease Control and Prevention, Chengdu, The People’s Republic of China; 4 Department of Environmental and Occupational Health, University of Colorado, Colorado School of Public Health, Aurora, CO, United States of America; Justus Liebig University Giessen, GERMANY

## Abstract

**Background:**

In areas where schistosomiasis control programs have been implemented, morbidity and prevalence have been greatly reduced. However, to sustain these reductions and move towards interruption of transmission, new tools for disease surveillance are needed. Genomic methods have the potential to help trace the sources of new infections, and allow us to monitor drug resistance. Large-scale genotyping efforts for schistosome species have been hindered by cost, limited numbers of established target loci, and the small amount of DNA obtained from miracidia, the life stage most readily acquired from humans. Here, we present a method using next generation sequencing to provide high-resolution genomic data from *S*. *japonicum* for population-based studies.

**Methodology/Principal Findings:**

We applied whole genome amplification followed by double digest restriction site associated DNA sequencing (ddRADseq) to individual *S*. *japonicum* miracidia preserved on Whatman FTA cards. We found that we could effectively and consistently survey hundreds of thousands of variants from 10,000 to 30,000 loci from archived miracidia as old as six years. An analysis of variation from eight miracidia obtained from three hosts in two villages in Sichuan showed clear population structuring by village and host even within this limited sample.

**Conclusions/Significance:**

This high-resolution sequencing approach yields three orders of magnitude more information than microsatellite genotyping methods that have been employed over the last decade, creating the potential to answer detailed questions about the sources of human infections and to monitor drug resistance. Costs per sample range from $50-$200, depending on the amount of sequence information desired, and we expect these costs can be reduced further given continued reductions in sequencing costs, improvement of protocols, and parallelization. This approach provides new promise for using modern genome-scale sampling to *S*. *japonicum* surveillance, and could be applied to other schistosome species and other parasitic helminthes.

## Author Summary

Schistosomiasis is a devastating tropical disease that affects more than 200 million people worldwide. Over the past several decades, transmission control strategies implemented in China have reduced the prevalence and morbidity of *Schistosoma japonicum* in many areas. Infections still persist, however, and it is therefore of great interest to determine the sources of recurring infections. Genetic analysis is a promising means to achieve this. Towards this aim, we conducted a pilot study to assess the feasibility of using high-throughput sequencing to assess the geographic distribution of schistosome genetic variants. Because DNA yields from miracidia, the most easily accessible life stage, are insufficient for high throughput sequencing, we first employed whole genome amplification to obtain sufficient quantities of DNA. We then employed a technique that reproducibly sequences the same fraction of a genome across numerous samples. We successfully sequenced 6-year old *S*. *japonicum* samples from Sichuan Province, China, easily and economically identifying tens of thousands of variable loci, a sufficient number to discriminate fine-scale population structure. Further population sampling will help answer important questions concerning the persistence of infections, the sources of new infections, and whether parasite populations have undergone incipient evolution of drug resistance.

## Introduction

In China, schistosomiasis has been reduced from approximately 12 million cases in the 1950s to approximately 100,000 cases in 2014, and public health officials are attempting to interrupt transmission of schistosomiasis nationwide [1–3]. The success of such programs has important implications for public health, as there are approximately 200 million people infected worldwide, and health impacts include anemia, impaired growth and cognitive development, and, in the case of *S*. *haematobium*, cancer [4–6]. We have studied schistosomiasis in Sichuan Province, China for the past decade, documenting the reemergence and persistence of infections in some areas despite aggressive control efforts [7–9]. These remaining pockets of schistosomiasis highlight gaps in our ability to prevent new infections and ultimately interrupt transmission.

Advances in genomic technology offer new opportunities to examine the sources of schistosomiasis infections. Combining new sequencing technologies with computational genomics, it is possible to evaluate parasite relatedness over small temporal and spatial scales, and to infer infection pathways. Such methods have been used to infer detailed transmission trees and dispersal pathways in viral outbreaks [10,11]. Application of these methods to schistosomiasis is complicated by the inaccessibility of adult worm pairs, the cost of sequencing, and the limited DNA available from miracidia, the most readily available life stage and progeny of adult worm pairs.

Over the past decade, methods for genotyping several dozen microsatellite loci (repeating sequences of 2 to 6 nucleotide base pairs) from a single miracidia have been developed for the three schistosomes of major human health importance [12–14]. Microsatellite genotyping has been used to answer questions about population structure of schistosomes over landscapes [15–18] and host species [16,19], as well as to evaluate changes in parasite diversity following chemotherapy [20,21]. However, the power to resolve detailed questions about the source and relatedness of schistosomes is limited by the number of loci tested; multi-locus genotypes based on a limited number of microsatellite loci may be the same in siblings and cousins by chance alone, and therefore indistinguishable from clonal individuals [22]. Genetic stratification of populations may also not be discernable. This makes it challenging to evaluate recent inbreeding and population bottlenecks that may be important to understanding parasite transmission in areas approaching elimination.

Genotyping methods that take advantage of next generation sequencing, such as double digest restriction site-associated DNA sequencing (ddRADseq) [23], offer a complementary approach to microsatellite methods [24], and can detect orders of magnitude more loci at costs that continue to decline. In ddRADseq, tens of thousands of loci are selected for sequencing by digestion with two restriction enzymes followed by enrichment for fragments in a desired range of lengths. Because the restriction sites are generally conserved, the same set of fragments tends to be obtained across individuals, allowing identification of variation at tens of thousands of orthologous loci. The large number of loci obtained, and the fact that each locus is sequenced at considerable depth across the entire locus make it possible to identify tens of thousands of variants that describe geographic differences in genetic variation at a fine scale. An additional advantage of ddRADseq is the ease of marker development relative to microsatellites, as the latter is based on a highly curated and specific set of loci that require locus-specific primer development and PCR amplification. Furthermore, the cost of high-resolution sequencing is declining rapidly as technology evolves, and may be appropriate for large-scale epidemiological studies in the near future [25]. Finally, the availability of reference genomes of all three major schistosome species [26–29] makes evaluation of the recovery of loci in the targeted range possible, allowing the technique to be fine-tuned for the reproducible recovery of a specific subset of loci in these species.

We have initiated a collaborative project that leverages advances in next-generation sequencing to better understand the dynamics of schistosomiasis infections in areas where infection is approaching elimination. Here we report the development of a ddRADseq technique that we applied to archived *S*. *japonicum* miracidia preserved on Whatman FTA cards. We demonstrate the utility of this approach in capturing a large amount of genetic variation information and show how this information might be used to reveal population structure at fine spatial resolution in this population. Our long-term goal is to use the method described here to evaluate pathways of schistosomiasis infection in pockets of residual transmission. We thus focused our efforts on developing a method that is efficient, reproducible and practical for field-collected samples–able to accommodate the limited DNA available in field collected samples and appropriate for samples that have been transported and archived without need of cold-chain storage for multiple years.

## Methods

### *In silico* digest

Restriction enzyme double digestions were performed *in silico* on the *S*. *japonicum* reference genome [27] downloaded from schistodb.net (SJC_S000000—SJC_S024939, 25,048 contigs in total) [30] to determine expected fragment locations and size distributions following digestion. We used a Perl script written in-house to determine the location of restriction enzyme cut sites and the size distribution of expected double-digested fragments (fragments containing one end cut by one restriction enzyme and another end cut by a second restriction enzyme). Fragments that contained transposable elements were masked out because these highly duplicated elements tend to share high sequence similarity, and distinguishing orthologous from paralogous fragments among highly duplicated sequences is problematic. The remaining fragments were used in comparison to experimental results to assess what proportion of fragments were recovered.

### Sources of *S*. *japonicum*

*S*. *japonicum* adult worm DNA was obtained from the Schistosomiasis Resource Center via BEI Resources, NIAID, NIH (pooled genomic DNA from adult male and female *Schistosoma japonicum*, Chinese Strain, NR-36066). According to BEI, the worms used as founders in this standard stock population were originally collected in Anhui Province, China in 1928 and augmented with a second Anhui isolate in 1977.

Archived field-collected samples of *S*. *japonicum* miracidia were collected from infected humans in Sichuan Province, China in 2010 as described elsewhere [9,31]. Briefly, participants were tested for infection using the miracidia hatching test and miracidia were collected from positive hatching tests. Miracidia were collected from the top of the hatching test flask, isolated using a hematocrit tube or Pasteur pipette drawn to a narrow bore with a flame, washed three times with autoclaved deionized water and placed on a Whatman FTA indicating card (GE) for long-term storage. Discolored spots appear on the card where the sample is dropped. After drying, cards were stored in a desiccator at room temperature. We selected 15 miracidia obtained from three humans in two villages located approximately 15 km apart for the work described here. The names and exact locations of these villages within Sichuan Province are not provided to maintain anonymity and promote candid reporting.

#### Ethics

The research involving human subjects was approved by the Sichuan Institutional Review Board, the University of California, Berkeley, Committee for the Protection of Human Subjects, and the Colorado Multiple Institutional Review Board. Participants provided written, informed consent. Anyone testing positive for *S*. *japonicum* was informed of their infection status and referred to the local anti-schistosomiasis control station for treatment.

### Whole genome amplification from miracidia

Miracidia contain on the order of 1–2 ng of DNA to start with, and field-collected specimens on Whatman cards may degrade over time, making direct application of ddRADseq on such samples problematic. Instead, whole genome amplification was applied to single miracidia on Whatman cards using isothermal (or multiple) strand displacement amplification [32]. This amplification strategy was chosen because previous studies have shown that whole genome amplification (using GenomiPhi; GE Healthcare) is capable of amplifying DNA for use in ddRADseq without detectable bias or introduction of mutations [33,34].

Individual miracidia were extracted from Whatman cards using a Whatman Harris 2mm micro-core punch (Whatman WB100029). Following excision, punches underwent five consecutive 5-minute washes. The first three washes consisted of 200 μL FTA purification reagent, and the final two washes consisted of 200 μL TE buffer. After the final wash, punches were left to dry for at least 1 hour at room temperature. Miracidia DNA was amplified directly from the punch using GenomiPhi V3 whole genome amplification kits (GE Healthcare Biosciences 25660124) following the manufacturer’s recommended protocol for amplification, with minor adjustments made to accommodate amplification from a 2 mm disk. Specifically, for miracidia, dried disks were transferred to an amplification tube containing 20 μL of 1x denaturation buffer. Tubes were incubated at 95°C for 3 minutes and then immediately placed on ice. Liquid from the tube was then added to individual amplification pellets provided in the kit, and allowed to dissolve the pellet for 10 minutes on ice. After gentle mixing, the liquid was transferred back to its original tube with the 2mm disk still present, and each amplification tube was then subjected to 90 minutes of amplification at 30°C, followed by enzymatic heat kill at 65°C for 10 minutes, and ending with a hold at 4°C.

### Library preparation and quality assessment

Adult worm DNA or whole-genome-amplified miracidium DNA was digested with two restriction enzymes, *PstI*-HF (New England Biolabs (NEB) R3140), a 6-cutter, and *Sau3AI* (NEB R0169), a 4-cutter, for eight hours at 37°C. Following digestions, DNA was purified via solid phase reversible immobilization (SPRI) using Axygen AxyPrep paramagnetic beads. The adult worm DNA was divided into eight replicate samples at this point in the process. A universal adaptor corresponding to the *Sau3AI* cut site and another adaptor corresponding to the *PstI*-HF cut site were then ligated to digested and purified DNA fragments. Adaptors contained unique molecular identifiers (UMIs; eight consecutive Ns prior to the ligation site that allow for PCR clone filtering), and a sample-specific 5 bp barcode on the adaptor corresponding to the *PstI*-HF cut site (see S1 Table for adaptor sequences). Samples were combined in equimolar ratios, cleaned via SPRI, and fragments between 300 and 600 bp (sizes reflect fragment size before adaptor ligation) were collected using a Pippin Prep 1.5% agarose gel (Pippin CDF1510). Size-selected fragments were PCR amplified with primers that add unique sequencing indexes that are required to multiplex multiple sample libraries per sequencing lane (see S1 Table for PCR primer sequences). PCR primers are designed to amplify only double-digested fragments, effectively reducing the number of off-target fragments in the size collection range to an extremely small percentage of clones. Following PCR, libraries were cleaned via SPRI and tested for size recovery. They were then pooled and prepared for sequencing by combining libraries in an equimolar ratio. DNA libraries were sequenced on an Illumina HiSEQ platform using 125bp single-end reads (miracidia), or on an Illumina MiSEQ using 75 bp paired-end reads (worm DNA).

### Initial quality filtering of SNPs and identification of microsatellite loci

Following sequencing, PCR clones were filtered out(based on UMI sequences) and reads were de-multiplexed into individual samples using the program Stacks [35]; 54.8% of all reads were filtered as PCR clones before de-multiplexing. Reads were mapped to the *S*. *japonicum* reference genome using bowtie2 [36]. Recovery of fragments was assessed in comparison to *in silico* digested fragments (see above) using a combination of custom Perl scripts and the intersect command from bedtools [37]. Fragments containing a substantial amount of sequence from repetitive elements and low-copy duplicates that were not eliminated in our *in silico* screening process would have been un-mappable; empirically, they amounted to about 15% of the reference fragments. Variant discovery and filtering were performed using the Genome Analysis Toolkit (GATK) [38–40] Haplotype Caller and other utilities of GATK. Different filters were applied to single nucleotide polymorphisms (SNPs) and indels. For SNPs, variants with a quality depth score less than two, mapping quality less than 22, or mapping quality rank sum score less than -20 were filtered out. For indels, only those with quality depth scores less than 2 were filtered out. We used PALfinder [41], custom Perl scripts, and bedtools [37] to identify microsatellite loci in the raw sequencing reads and VCF files. A microsatellite locus was considered to be entirely located within a read if both its beginning and end were 10 or more bp away from the ends of where the read aligned to the reference genome.

### Population genetic analyses

We also applied more stringent filtering of recovered ddRADseq loci and SNPs to test our ability to use miracidia-derived data to make precision inferences about population structure and genetic variation. This filtering was aimed at ensuring that there were a sufficient number of high-quality reads for each locus to confidently discriminate heterozygous and homozygous calls at polymorphic sites. For these analyses, the forward read (i.e., read 1) for each parsed individual was first quality trimmed using the program Trimmomatic v. 0.33 [42] with the settings LEADING:10, TRAILING:10, SLIDINGWINDOW:4:15, and MINLEN:36. Quality-trimmed reads were mapped to the *S*. *japonicum* genome [27] using the MEM algorithm of BWA v. 0.7.15-r1140 [43], with shorter split hits marked as secondary (-M flag engaged). The radcap software package [44], which incorporates SAMtools v. 1.3 [45], Picard Tools v. 1.106 (http://broadinstitute.github.io/picard), and GATK [38–40], was used to perform the following: merge mapping files, realign around indels, call variants using the Unified Genotyper (both SNPs and indels), and filter SNPs around indels and by quality (genotype calls with a read depth < 5 and a quality score < 20 were removed).

Only SNPs were used for subsequent analyses. A custom script was used to remove SNPs called against the genome that were either monomorphic in our samples or non-biallelic, and to code individual genotypes as missing data if the genotype quality score was below 20 or the individual read depth fell below 10x. Lastly, variants were filtered out using VCFtools v. 0.1.15 [46] to control the number of samples missing data at each locus (i.e., samples that did not have sufficient data mapped at a locus to pass the more stringent quality filters). This resulted in three datasets: one with all loci that were not missing data in any of the eight samples (one adult worm sample and eight miracidia samples); one that excluded the adult worm sample and included all loci that were not missing data in any of the miracidia; and one that excluded the adult worm sample and allowing missing data from two of the eight (25%) miracidia. This last sample was aimed at discovering loci that might have incomplete representation but sufficient sample representation to be of utility in some cases.

Custom Perl scripts were used to calculate the proportion of heterozygous (or polymorphic) loci among variable loci for each sample in the three data sets. RAxML 8.0 [47] was used to infer a maximum likelihood phylogenetic tree detailing relationships based on SNP variation in the nine-sample dataset. For RAxML analyses, we applied an ascertainment bias correction because our SNP collection contained no invariant sites; we otherwise used the default program settings, and specified 1,000 bootstrap replicates following the ML search. While we did not pursue it here, we note that using the full set of ddRADseq loci instead of only variable sites can produce even more accurate tree estimates, which is relevant for extending the utility of this type of data [48]. Principle components analysis (PCA) using the R package SNPRelate [49] was applied to the nine-sample dataset with and without the adult worm data. A custom Perl script was used to calculate pairwise genotype sharing among samples. Briefly, genotypes at every locus were compared between samples and determined to be either 100% identical, 50% similar, or 0% similar, and the mean similarity was calculated. Variation in similarity was calculated from 1,000 permutations, sampling variants at random with replacement.

### Data availability

Variant datasets, microsatellite information, and custom scripts are deposited in the Dryad repository: http://dx.doi.org/10.5061/dryad.8091q [50]. Sequences have been deposited in the NCBI Sequence Read Archive and can be accessed through BioProject ID PRJNA349754.

## Results

### Correspondence between *in silico* and empirical results

**Fig 1:**
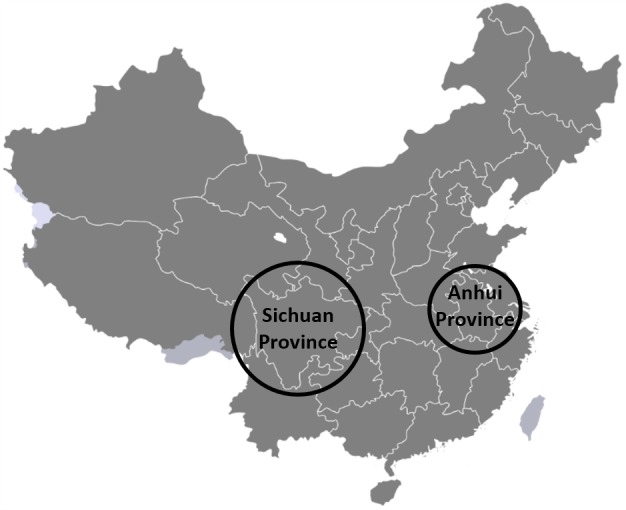
Origin of samples. This map of China shows the two provinces sampled. Village locations are not shown to maintain anonymity. The miracidia were sampled in Sichuan Province. The original source of the Chinese Strain from which the adult worm sample was pooled was Anhui Province.

**Fig 2:**
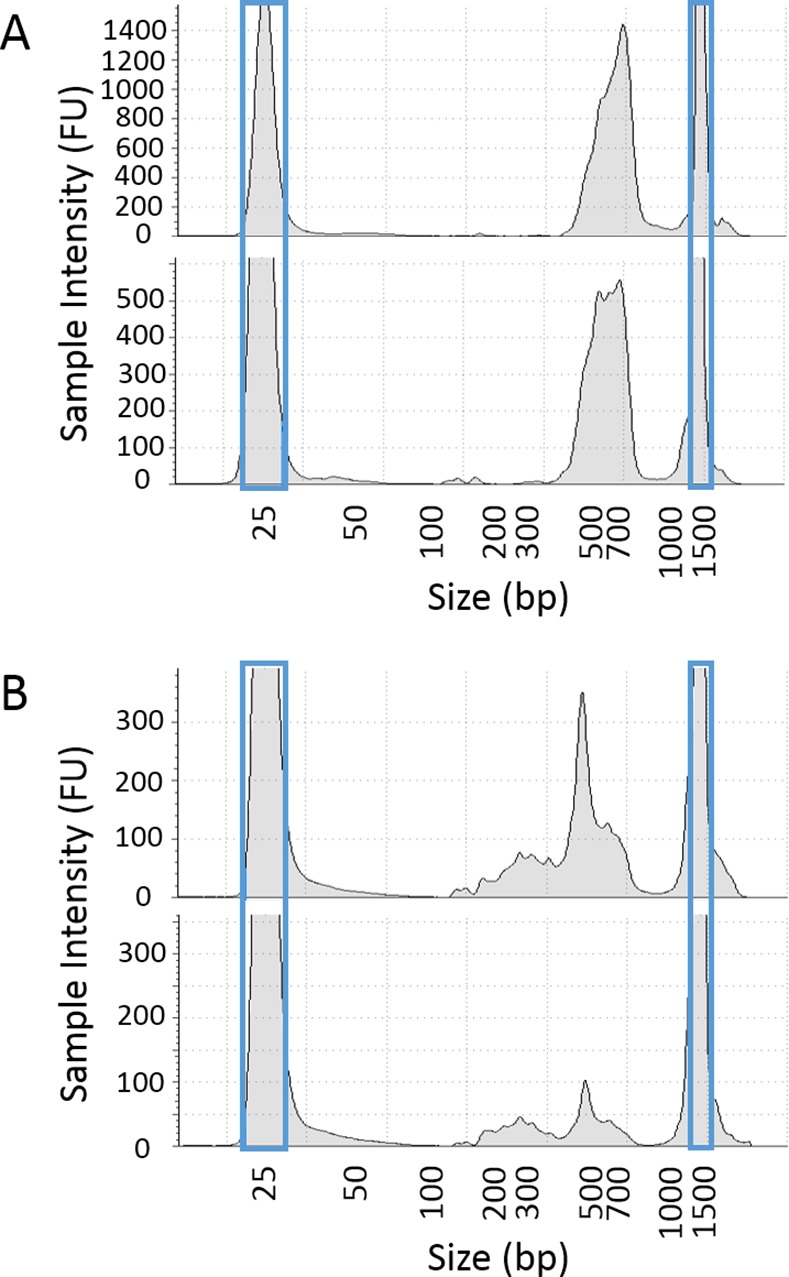
Distribution of genome library sizes. The distribution of library fragment sizes obtained after size selection and amplification are shown in fluorescence units (FU), as measured on an Agilent TapeStation. The results for the two libraries created from (a) the unamplified adult worm DNA, and (b) the results for the two libraries created from the genome-amplified six-year old miracidia samples. Blue boxes depict the location of lower and upper markers used as standards for sample measurements. Note that the scales of the y-axes are not consistent between graphs.

**Fig 3:**
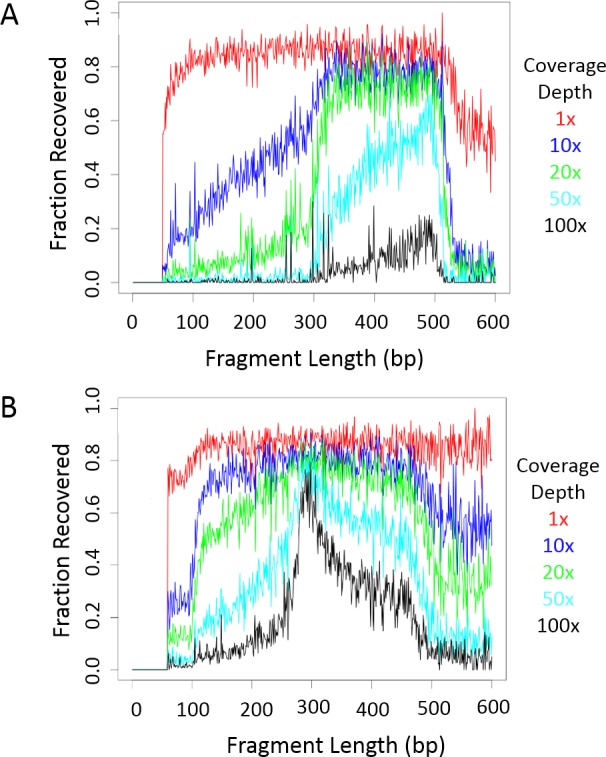
Recovery of loci in DNA sequences. (a) The fraction of expected genomic fragments recovered in a single representative replicate (index 1, tag 1) of unamplified adult worm DNA is shown for each fragment size at different coverage depths. All eight replicates produced similar results (S2 Fig). (b) The fraction of expected genomic fragments recovered in a single sample (index 1, tag 1, miracidia 5) of amplified miracidia is shown for each fragment size up to 600bp at different coverage depths. S3 Fig displays results for all eight samples.

Based on comparisons of *in silico* digestions of the complete genome sequence of *S*. *japonicum* [27] using different potential pairs of restriction enzymes, we chose the combination of *PstI*-HF and *Sau3AI* for empirical ddRADseq library construction and sequencing. This pair of restriction enzymes was predicted to produce 17,131 double-digested fragments in the 300–600 bp range that would map to unique regions in the genome (S1 Fig). For reference, these fragments comprise 7.29 Mbp of the 397 Mbp in the *S*. *japonicum* genome [27]. To benchmark the ddRADseq approach on high-quality DNA, we first tested it on Chinese Strain adult worm DNA from Anhui Province (Fig 1). We ran eight replicates post-digestion (pre-ligation), using different barcode and index combinations to test that each adaptor worked and that we could recover the same loci across experiments. The size-selected and amplified libraries contained fragments mostly in the 450–700 bp range (including 143 bp adaptors and amplification primers), indicating that our size targeting and purification procedure was largely successful, but with some loss of longer fragments (Fig 2A). The pooled replicate libraries were sequenced to obtain a total of over 38 million paired-end reads that were then mapped to the reference genome. Recovery of *in silico* expected loci across a range of sequencing depths was generally at most 85%, indicating that about 15% of the expected loci either did not doubly digest or were un-mappable due to repetitive elements or low-copy duplicates not eliminated by the *in silico* screening process. Recovery in the 300–500 bp range was excellent, with about 70% of loci (out of 85% maximum) sequenced at least 20x in each replicate (Fig 3A and S2 Fig). Fragment lengths longer than 500 bp are not well represented, probably due to biases in amplification and recovery, but this is compensated by a reasonable recovery rate for sequences in the 100–300 bp range.

To test the ddRADseq method on field-collected samples, we performed whole genome amplification on miracidia samples from three human individuals that had been archived on Whatman cards in 2010. Amplification was successful from 13 out of 15 miracidia, with an average production of approximately 6 μg of DNA. Eight of the 13 were subjected to multiplexed ddRADseq library preparation and pooled; approximately two thirds of the DNA in the eight sequenced miracidia libraries was in the target size range, while the remaining third was in the 150–450 bp range (including adaptors and primers, Fig 2B). This excess of short off-target DNA is common for genome amplification, partly because more PCR amplification cycles are required to obtain sufficient DNA for quantification and sequencing.

**Fig 4:**
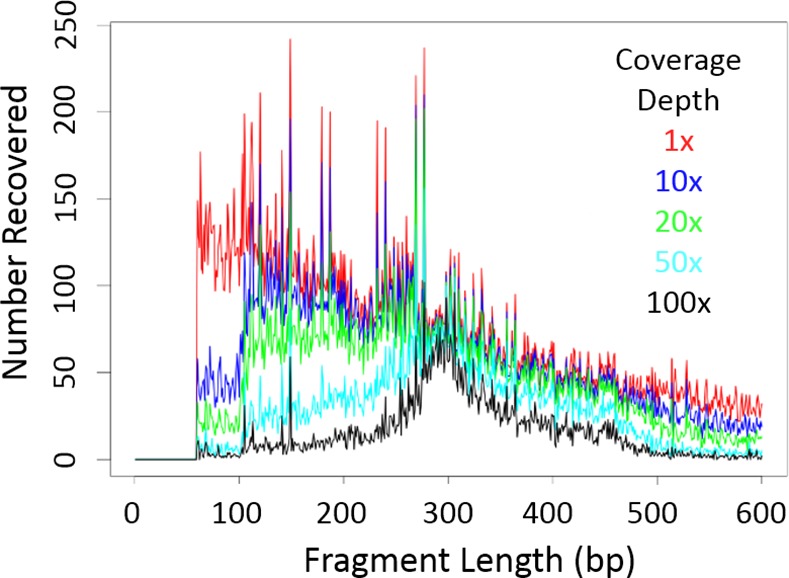
Number of loci recovered among miracidia samples. The number of genomic fragments recovered in a single sample (index 1, tag 1, miracidia 5) of amplified miracidia is shown for expected fragment sizes up to 600bp at different coverage depths. The results were similar among miracidia samples (S4 Fig).

The pooled miracidia libraries were sequenced to obtain a total of over 280 million 125 bp single-end reads. Nearly all reads were generated from RAD double-digested fragments; 28.48 million reads (98.5%) that map to unique regions in the *S*. *japonicum* reference genome begin with the expected restriction site sequence. Recovery of fragments in the 300–500 bp range at 20x coverage was comparable to the worm replicates (about 70% out of a maximum of 85%) for most samples, but as expected based on the library length distribution, more recovered sequences mapped to shorter fragments than in the case of the un-amplified worm (Fig 3B and S3 Fig). This means that more loci provided good sequence and variation data than were enriched for using size selection. For example, in one of the sequenced miracidia (Fig 4 and Table 1) there were 10,899 loci covered at 20x or more in the 300–600 bp range, and 26,794 < = 600 bp; at 10x or more coverage, it had 32,804 loci < = 600 bp. Similar results were obtained from other miracidia (S4 Fig and S2 and S3 Tables).

**Table 1:** Number of fragments sequenced from a single miracidium (index 1, barcode 1) at different depths in different size ranges. Results were similar across the eight miracidia sequenced.

	Size Range	
Coverage	300–600 bp	≤ 600 bp
1x	14,843	41,406
10x	12,602	32,804
20x	10,899	26,794
50x	7,695	16,130
100x	4,751	8,853

### Microsatellite identification

Though not the primary motivation of our study, the large sample of *S*. *japonicum* loci enabled us to identify many microsatellite loci that may be of use in future population genetics studies. We located 33,286 paired end reads with microsatellite loci in the adult worm data. Forward and reverse PCR primers were designed for these loci, and after filtering out duplicated sequences and primers, 1,609 unique primer sets remained (S4 Table); we were unable to assess indel variation at these loci because the microsatellite regions generally overlapped with the unsequenced regions between the paired ends.

To evaluate the utility of ddRADseq for direct microsatellite genotyping and microsatellite/SNP comparative analysis, we determined that 11,208 microsatellite loci were sequenced at any depth in at least half of the eight miracidia samples. 7,595 of these microsatellite loci were wholly contained within the sequence read, indicating their potential for microsatellite genotyping, and 1,100 of them were variable in the microsatellite region among the eight sequenced samples. Well over half of the variable loci (652, or 59%) passed low stringency variant filtering (see Methods) in all eight miracidia samples, and 123 of these had indel variants in the microsatellite loci. Although this amount of variability is low for microsatellites in general, it is perhaps reasonable given the bias towards short loci caused by the requirement that they be fully contained within the 125 bp sequencing read. Even so, this represents an approximately seven-fold increase in indel information from the 17 microsatellite loci used in previous *S*. *japonicum* PCR studies [12,13].

### Heterozygosity and fine-scale population structure in SNP variants

There were 25,721 SNP variants that passed stringent quality and coverage filters for all nine samples. The average proportion of heterozygous (or polymorphic, for the adult worm sample) SNPs among these samples was 0.29 (s.d. 0.03), which is over 438 times as many variable loci as would maximally be obtained from previous PCR-based microsatellite studies in *S*. *japonicum*. It was somewhat surprising to us that the proportion of polymorphic loci in the adult worm sample is 0.33 because it comes from a strain that has been maintained for nearly 90 years, with the addition of only a single outside isolate nearly 40 years ago. The high amount of variation is somewhat assisted by the fact that it comes from a pooled sample and the fact that with only nine samples we are mostly considering high frequency SNPs, but it is also a testament to sound strain maintenance practices that must have avoided severe population bottlenecks. The genome-amplified miracidia samples appear to contain many more off-target short loci than the unamplified adult worm data, and the filtering requirement that all variant loci be genotyped in all samples excludes from analysis many variants found in everything but the adult worm. We therefore created a dataset of SNP variants that passed stringent quality and coverage filters for just the eight miracidia samples; this dataset contained 67,525 variants, about 2.5 times the number that were also shared with the adult worm sample. We were also interested to identify loci that might commonly but not always provide data; we therefore created a third dataset including loci that were missing quality data in up to two of the eight miracidia, and obtained 102,877 variants, or about four times the number of variants than shared with all nine samples including the adult worm sample. There was more variation in this sample, with an average heterozygosity of 0.37 (s.d. = 0.04), which is over 2,000 times as many variable loci as could be obtained from previous microsatellite PCR studies in *S*. *japonicum*.

**Fig 5:**
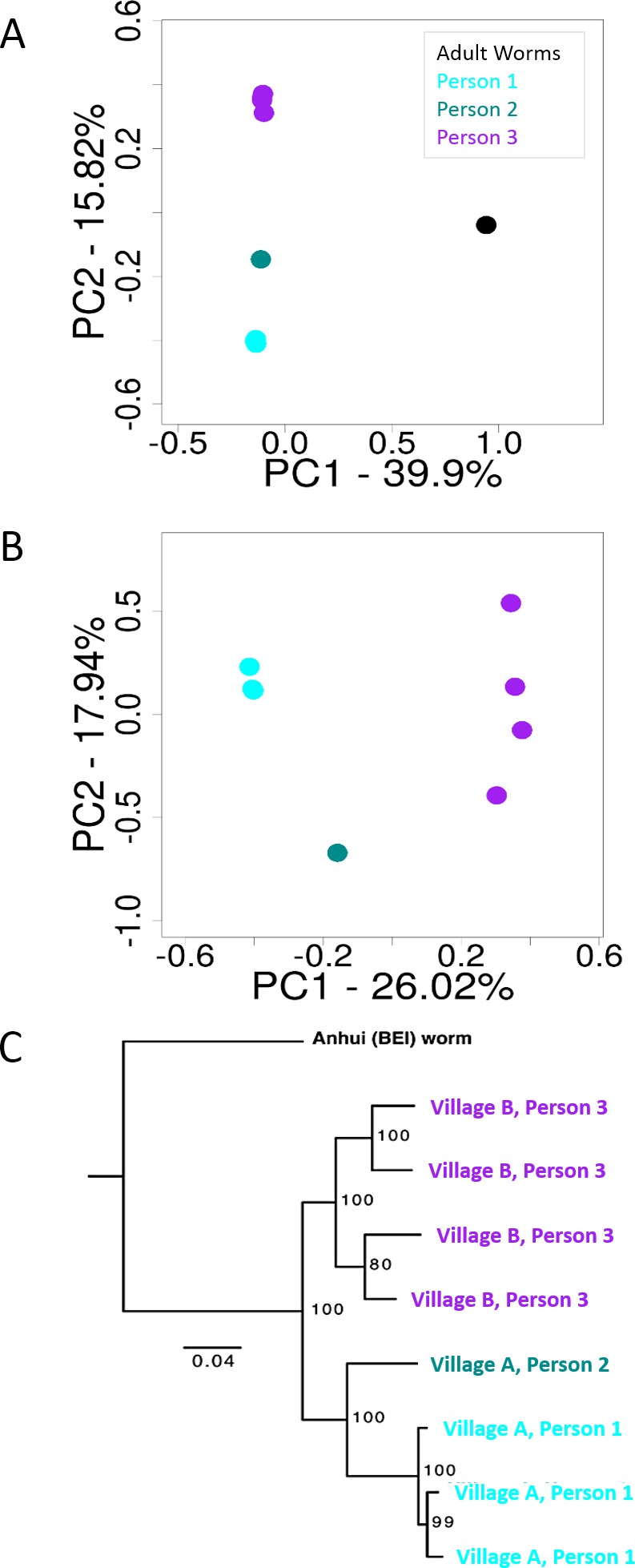
Principle components analysis of *Schistosoma* samples. Principal component analysis from (a) bi-allelic SNPS called in all nine samples, and (b) all eight miracidia samples. Note that there are three miracidia from Person 1 and four miracidia plotted from Person 3 in both (a) and (b), though their proximity to each other obscures the multiple datapoints. (c) Maximum likelihood phylogeny inferred using ddRADseq data showing genetic structure and relationships among *Schistosoma* samples.

Although this study was not specifically designed to test population structure because of the limited population sampling included here, we conducted principle components analysis [51,52] on the nine-sample shared SNP dataset to obtain a preliminary estimate of how much this massive increase in genetic variation information might enable the discrimination of population structure in future studies. The first principle component was almost entirely devoted to separating the Anhui adult worm stock sample from the Sichuan miracidia samples, with the second principle component mostly separating the miracidia depending on which person they came from (Fig 5A). We interpret this result with caution because the adult worm sample and miracidia samples were prepared differently (see Methods), and the adult worm sample from a stock strain is unlikely to reflect current Anhui isolates; however, it is clear that genetic differences between the adult worm and miracidia samples are much greater than differences among miracidia. To test if we could obtain additional resolution from the larger dataset excluding the adult worm sample, we ran an additional PCA with the dataset excluding the adult worm sample and found that the first principle component cleanly separated the miracidia obtained from different people and from different villages. The second principle component tended to separate different samples within people, although the miracidia within Person 1 were still not well differentiated (Fig 5B). In summary, although the limited sample size precludes a more in-depth analysis of population structure, the amount of highly informative genetic variation obtained does enable the detection of clear genetic differences between miracidia samples.

To complement the PCA analyses, we inferred a phylogenetic tree using the stringent set of SNPs in all nine samples (Fig 5C). There was nearly 100% bootstrap support for all but one clade on the tree (which had 80% bootstrap support), with the first major split separating the miracidia (all from Sichuan Province, Fig 1) and the adult worm (from Anhui Province stock, Fig 1). While we interpret this result with caution due to the Anhui sample representing a long-standing and previously supplemented stock sample, it is notable that our result is consistent with previous results indicating genetic separation between provinces [53]. The miracidia from each village formed strongly supported clades, as did the miracidia from human 1 within village A. The four samples from a single human host from village B exhibited considerably more structure and longer branch lengths compared to the three samples from a single human host from village A. These results generally mirror and support those from the PCA analyses, and suggest that even with this limited sample, the large amount of variation information is sufficient to clearly identify patterns that distinguish *S*. *japonicum* from different provinces, villages, and people. Because the size of the sample is so small, we could not get accurate estimates of allele frequencies, and thus could not make estimates of relatedness definitive enough to qualify as results; however, we include the implications of preliminary estimates of relatedness in the discussion below.

## Discussion

We have shown here that *S*. *japonicum* miracidia samples archived for many years at room temperature on Whatman cards can be used to provide large amounts of valuable individual differentiation and population structure information for relatively low cost. We were able to consistently and economically obtain sequence information from tens of thousands of loci, yielding approximately 100,000 SNPs genotyped from multiple miracidia samples, even with stringent filtering criteria. We showed that although ddRADseq locus recovery varies among samples (see Figs 3, S2, 4 and S4), reproducible and in-depth recovery of the majority of genomic fragments from a selected size range is possible, even between libraries prepared from different source DNA types, and with different sequencing protocols. It is this reproducible recovery of a limited number of loci across samples without the need for extensive protocol optimization that gives ddRADseq much of its power.

In addition to surveying genome-wide SNPs, we also identified approximately 11,000 microsatellite loci, about 10% of which had quantifiable variation among the eight miracidia sequenced and 123 of which contained indel variation. Furthermore, we designed PCR primers for 1,609 new microsatellite loci that could be used alone or in conjunction with high-throughput sequencing experiments—this resource provides a considerable increase from the 17 microsatellite loci used previously to genotype *S*. *japonicum* samples [31]. Importantly, this study demonstrates the ability to obtain many orders of magnitude more data than was previously possible, even from archived miracidia samples collected six years in the past. This drastically expands the ability to evaluate parasite population dynamics through time and space.

A useful aspect of the ddRADseq method is that it is flexible enough to handle different sample types while remaining relatively easy to scale the amount of data collected per sample to balance cost and accuracy requirements. This can be done by changing the restriction enzymes and/or by changing size-selection of fragments to include greater or fewer loci to target per sample. Such alterations of the protocol, coupled with adjustments to the numbers of samples pooled per sequencing run, allow the approach to be readily re-scaled to address particular questions. For example, by combining an extended set of the adaptor barcodes and PCR-added indices utilized in this study, and size-selecting a smaller set of loci, the approach could readily be scaled to include over 100 samples per Illumina HiSEQ sequencing lane. Furthermore, such high multiplexing of samples, coupled with modern SNP calling approaches that incorporate uncertainty in SNPs (genotype uncertainty methods; [54]), allow for a high degree of parallelization and economy without sacrificing accuracy in SNP calling. Using such approaches one can obtain useful data even from regions or samples with less-than-ideal sequencing coverage. In our laboratory, costs per sample were in the $50-$200 range, depending on the amount of sequence information produced per sample. In addition to parallelization, we expect that sequencing costs will decrease and that protocols can be refined or improved, further reducing costs. Nevertheless, our results demonstrate that it is possible to affordably obtain large amounts of variation using ddRADseq without excessive protocol optimization. This technique therefore promises to be both a powerful and a cost-effective tool in the arsenal against neglected tropical diseases.

Variant filtering strategies for studies with different purposes may vary substantially from the variant filtering we performed here, depending on the goals and tolerances of a particular analysis. We applied both low and high stringency filters to allow the possibility of interpreting all variants with a probabilistic approach and making use of as much of the data as possible, as well as to provide for more traditional (non-probabilistic) direct estimates of relatedness and genetic variation. The flexibility of ddRADseq readily enables adjustments to be made to the amount of sequencing per sample as appropriate for the questions being addressed. For example, high confidence variant calling applications, such as association studies, may require higher coverage, and thus need relatively more sequencing. Analysis of gross population structure or sibling detection, in contrast, may require less coverage depth per locus, and may produce better results if sequencing efforts are focused on more multiplexing and larger sample sizes.

The ability to reliably sequence large numbers of loci from numerous archived miracidia at economic costs enables the use of this method to answer a number of epidemiological questions relevant to the control of *S*. *japonicum* using field-collected samples. With increased sampling within and among human individuals, for example, it may be possible to answer questions such as: 1) How many genetically distinct adult worm mating pairs are active in an individual? 2) Is there evidence that an individual who is repeatedly infected is harboring the same adult worm pair, or are they repeatedly infected with new worms? 3) Do infections in geographically-clustered individuals appear to come from a single source, and how are such individuals geographically distributed? 4) Do infections in a single village tend to come from a restricted local source, or are they acquired from a larger region? 5) What proportion of human schistosomiasis burden can be attributed to non-human mammalian reservoirs?

Finally, although we could not obtain accurate estimates of allele frequencies due to the small and non-random sample size in this pilot study, it is of interest to discuss preliminary estimates of relatedness obtained from this data to demonstrate the potential to apply this type of data in a broader epidemiologically-relevant context with far greater sampling. We made such preliminary estimates by calculating genotype sharing between individual miracidia, which can be estimated with low variance from this data (S5 Table). The amount of genotype sharing between miracidia sampled in different villages is 0.75–0.79, and there is one miracidia pair within each village that is also in this range. For the sake of discussion, we assume, despite the small sample size and proximity of the villages, that this is the range of sharing we might expect among unrelated individuals or distant cousins within a Province. At the other extreme, the miracidia from Person 1 share a substantially higher proportion of variants than other pairs (0.87–0.90, S5 Table); this is consistent with the idea that they are all siblings, progeny of the same adult worm pair or progeny of clones of the same pair, and is consistent with the PCA and phylogenetic results. Sibling relationships are a reasonably likely outcome if this person was infected by a single mating worm pair at the time of sampling, although the amount of sharing is slightly high if the parents were unrelated, indicating that the parent worms may have been cousins. It is also possible that the higher genotype sharing in one pair (miracidia 1 and 3, sharing = 0.90) indicates that they are full siblings, while the other two pairs (miracidia 1 and 2, and 2 and 3, sharing = 0.87) are half siblings with related half-parents. We plan to further evaluate this hypothesis with better population sampling and allele frequency estimates, but it is of epidemiological interest because it potentially indicates a mixture of clones and non-clones among the parental worm pairs.

The genotype sharing for two of three miracidia pairs from different people in the same village (Person 1 and Person 2 from Village 1, comparing miracidium 4 versus miracidia 1 and 3) is compatible with the idea that their miracidia show 2^nd^ or 3^rd^ degree (close cousin) relatedness, as do three of the four miracidia from Person 3. The relatedness of these pairs are in the range of 0.80–0.83, somewhat above the average 0.77 in presumed unrelated individuals, but substantially below the average 0.88 in the presumed siblings from Person 1. This suggests either considerable local geographic population structure or a higher probability of cousin relationships within villages. If these preliminary results are supported by more in-depth study, they may indicate that sources of infection are village specific. If widespread evidence of 2^nd^ or 3^rd^ degree relatedness among and within people in the same village is confirmed, this would suggest that infections in villages may be the progeny (offspring or grand-offspring) of an extremely limited number of adult worm pairs (which could live in a human or non-human reservoir). A more in-depth analysis using complete haplotype sets between worm pairs would likely be fruitful, though we note that this analysis is not yet feasible due to the fragmented nature of the reference genome and small population sample size in this study.

Given its prevalence and the serious health risks it poses, and in light of the considerable efforts to attain regional elimination of *S*. *japonicum* in China, it is essential to develop practical genomic tools that are capable of resolving complex questions of schistosome transmission to assist complete elimination in China and extend such success to other countries and schistosome species. The ddRADseq-based genotyping method applied here is appropriate for field collected samples: it is able to accommodate the limited DNA available in schistosome miracidia, and appropriate for samples that have been stored for multiple years in a format that does not require refrigeration, allowing easy transport from the field to the laboratory. This method should enable detailed determination of population structure that can be used to accurately identify sources of infections and reinfections, creating the potential to track and target human or mammalian source reservoirs. Finally, we expect that the ddRADseq variation information can be used to identify genetic adaptation events in these parasitic worms, and thus enable early detection and eradication of strains that may evolve resistance to the critical anthelmintic drug Praziquantel. Given the unique role of this drug in schistosome control in Asia as well as worldwide, such detection may prove invaluable to prevent a great deal of future human suffering.

## Supporting Information

S1 Fig Predicted fragment size distribution from double restriction enzyme digestion. The predicted distribution of double-digested fragment (those with one cut site from each enzyme) sizes is shown, as determined by *in silico* digestion of the reference *S*. *japonicum* genome [27].
(TIF)

S2 Fig Recovery of loci in worm DNA sequences. The fraction of expected genomic fragments recovered in a single replicate from unamplified adult worm DNA is shown for each fragment size at different coverage depths. Individual samples are depicted as follows (a) index 1, barcode 1; (b) index 1, barcode 2; (c) index 1, barcode 3; (d) index 1, barcode 4; (e) index 2, barcode 1; (f) index 2, barcode 2 (g) index 2, barcode 3; (h) index 2, barcode 4. Note that (a) is the same figure shown in Fig 3A.
(TIF)

S3 Fig Recovery of loci in miracidia DNA sequences from eight samples. The fraction of expected genomic fragments recovered from each of the amplified miracidia is shown for each fragment size up to 600bp at different coverage depths. Individual samples are depicted as follows (a) index 1, barcode 1, miracidia 5; (b) index 1, barcode 2, miracidia 6; (c) index 1, barcode 3, miracidia 1; (d) index 1, barcode 4, miracidia 2; (e) index 2, barcode 1, miracidia 3; (f) index 2, barcode 2, miracidia 7; (g) index 2, barcode 3, miracidia 8; (h) index 2, barcode 4, miracidia 4. Note that (a) is the same graph shown in Fig 3B.
(TIF)

S4 Fig Number of loci recovered among eight miracidia samples. The number of genomic fragments recovered from each of the amplified miracidia is shown for expected fragment sizes up to 600bp at different coverage depths. Individual samples are depicted as follows (a) index 1, barcode 1, miracidia 5; (b) index 1, barcode 2, miracidia 6; (c) index 1, barcode 3, miracidia 1; (d) index 1, barcode 4, miracidia 2; (e) index 2, barcode 1, miracidia 3; (f) index 2, barcode 2, miracidia 7; (g) index 2, barcode 3, miracidia 8; (h) index 2, barcode 4, miracidia 4. Note that (a) is the same graph as shown in Fig 4.
(TIF)

S1 Table ddRADseq adaptor and primer sequences. Sequences of olionucleotides used as adaptors and PCR primers.
(PDF)

S2 Table Number of fragments sequenced between 300 and 600 bp recovered in each miracidium sample. Index barcode combinations correspond to individual miracidia as follows: index 1, barcode 1, miracidia 5; index 1, barcode 2, miracidia 6; index 1, barcode 3, miracidia 1; index 1, barcode 4, miracidia 2; index 2, barcode 1, miracidia 3; index 2, barcode 2, miracidia 7; index 2, barcode 3, miracidia 8; index 2, barcode 4, miracidia 4.
(PDF)

S3 Table Number of fragments under 600 bp sequenced in each miracidium sample. Index barcode combinations correspond to individual miracidia as follows: index 1, barcode 1, miracidia 5; index 1, barcode 2, miracidia 6; index 1, barcode 3, miracidia 1; index 1, barcode 4, miracidia 2; index 2, barcode 1, miracidia 3; index 2, barcode 2, miracidia 7; index 2, barcode 3, miracidia 8; index 2, barcode 4, miracidia 4.
(PDF)

S4 Table PCR primers for potentially amplifiable microsatellite loci. Primer sequences, annealing temperatures, and related information for potentially amplifiable microsatellite loci from paired-end sequencing of *S*. *japonicum* ddRADseq loci.
(XLSX)

S5 Table Genotype sharing among eight miracidia samples. Pairwise comparison of similarity between 8 miracidia samples at 67,525 bi-allelic variants. The mean similarity is depicted as the top number in each cell, with the mean ± 2 standard deviations shown as the bottom row in each cell. Shading within cells corresponds to the degree of similarity for the two miracidia being compared, with darkers shades of gray indicating more similarity.
(PDF)
