# Supplementary figures and images for: Whole Genome Amplification and Reduced-Representation Genome Sequencing of Schistosoma japonicum Miracidia
Source: PLoS Negl Trop Dis. 2017 Jan 20;11(1):e0005292. doi: 10.1371/journal.pntd.0005292 (PMC5287463; doi:10.1371/journal.pntd.0005292)

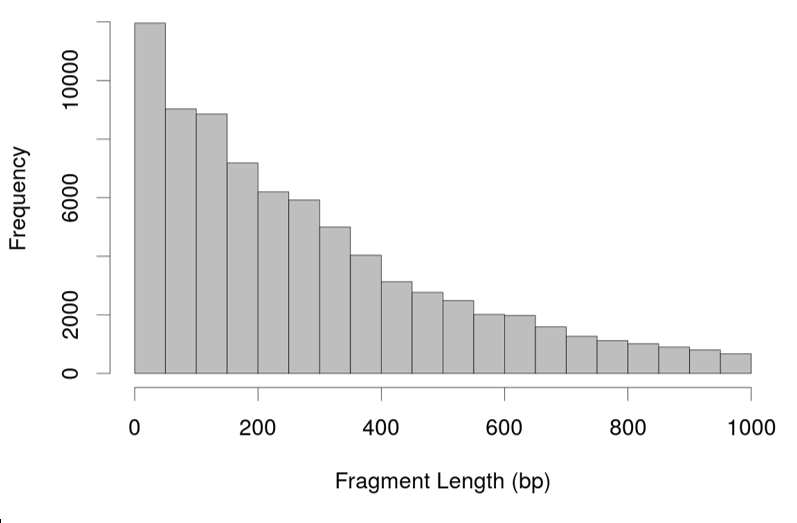

Supplement: S1 Fig — The predicted distribution of double-digested fragment (those with one cut site from each enzyme) sizes is shown, as determined by in silico digestion of the reference S. japonicum genome [27]. (TIF) [file pntd.0005292.s001.tif]

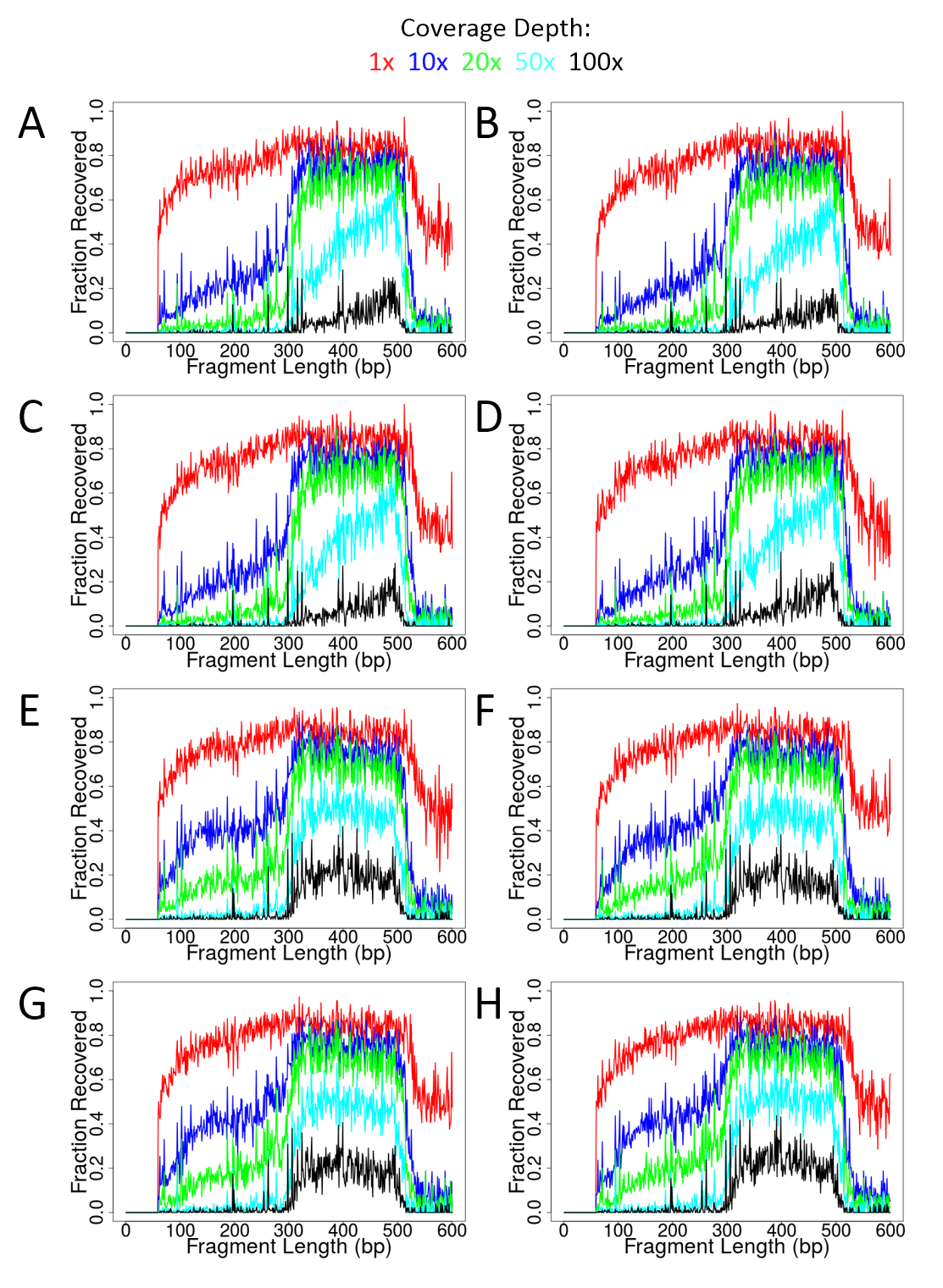

Supplement: S2 Fig — The fraction of expected genomic fragments recovered in a single replicate from unamplified adult worm DNA is shown for each fragment size at different coverage depths. Individual samples are depicted as follows (a) index 1, barcode 1; (b) index 1, barcode 2; (c) index 1, barcode 3; (d) index 1, barcode 4; (e) index 2, barcode 1; (f) index 2, barcode 2 (g) index 2, barcode 3; (h) index 2, barcode 4. Note that (a) is the same figure shown in Fig 3A. (TIF) [file pntd.0005292.s002.tif]

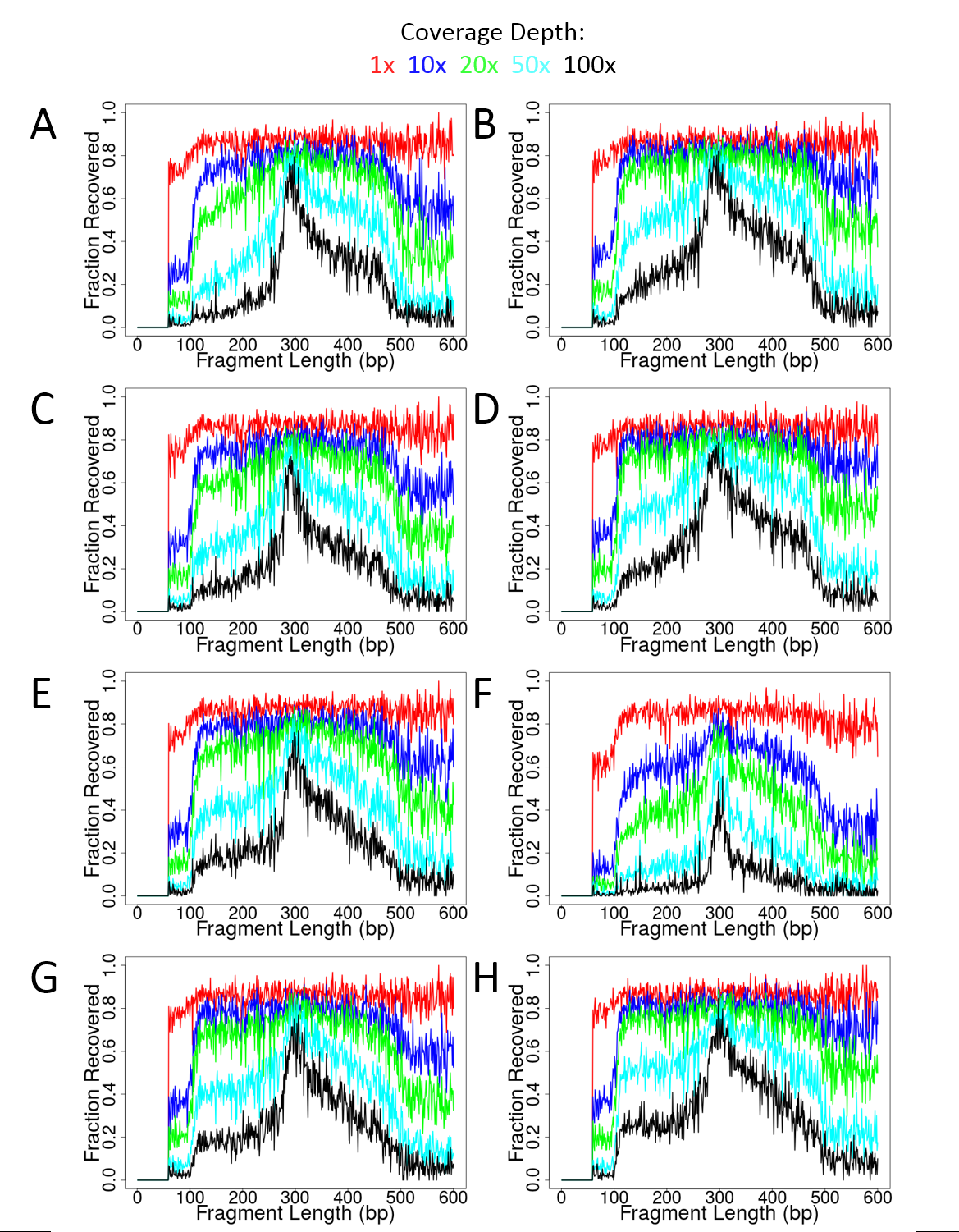

Supplement: S3 Fig — The fraction of expected genomic fragments recovered from each of the amplified miracidia is shown for each fragment size up to 600bp at different coverage depths. Individual samples are depicted as follows (a) index 1, barcode 1, miracidia 5; (b) index 1, barcode 2, miracidia 6; (c) index 1, barcode 3, miracidia 1; (d) index 1, barcode 4, miracidia 2; (e) index 2, barcode 1, miracidia 3; (f) index 2, barcode 2, miracidia 7; (g) index 2, barcode 3, miracidia 8; (h) index 2, barcode 4, miracidia 4. Note that (a) is the same graph shown in Fig 3B. (TIF) [file pntd.0005292.s003.tif]

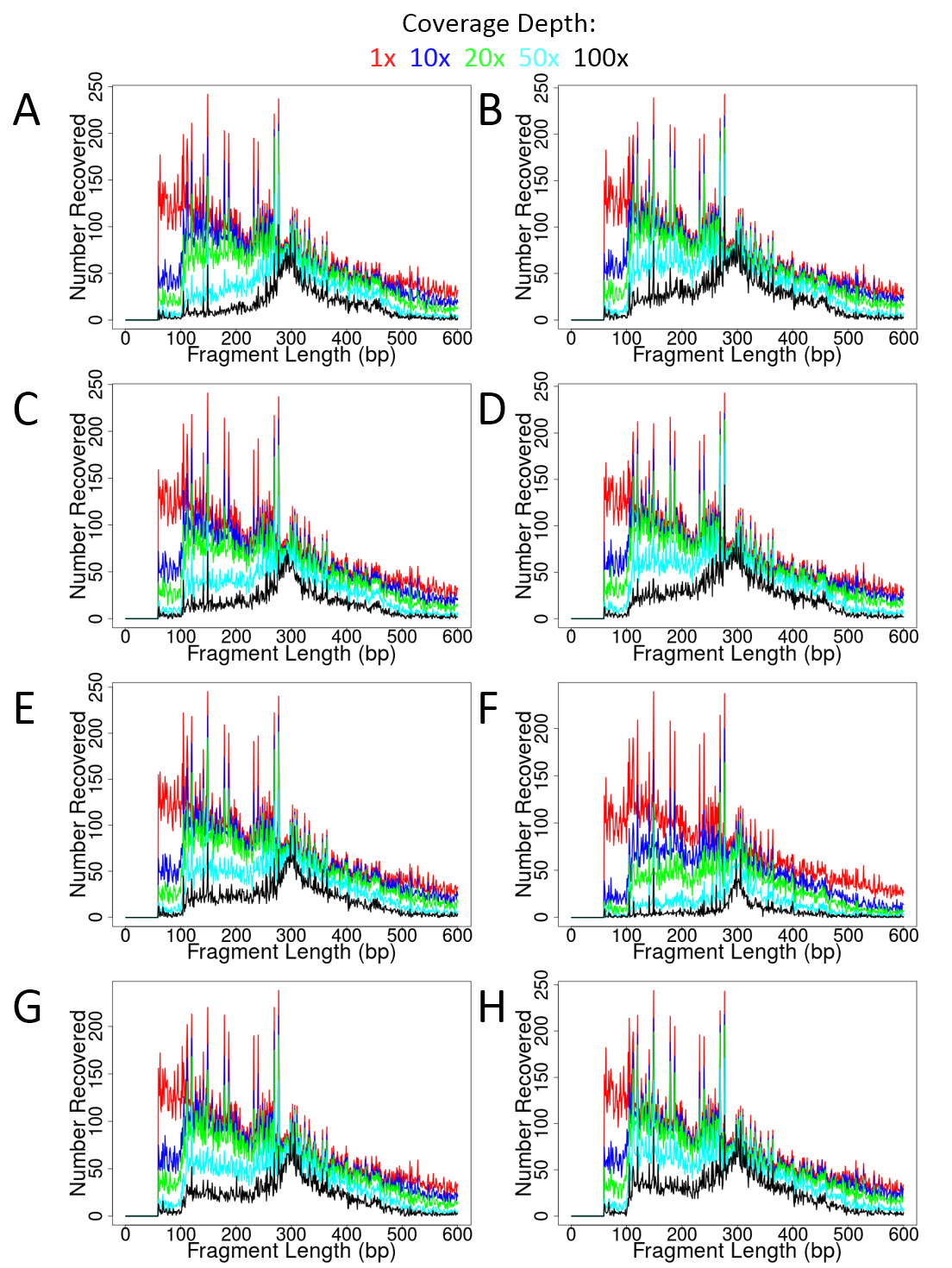

Supplement: S4 Fig — The number of genomic fragments recovered from each of the amplified miracidia is shown for expected fragment sizes up to 600bp at different coverage depths. Individual samples are depicted as follows (a) index 1, barcode 1, miracidia 5; (b) index 1, barcode 2, miracidia 6; (c) index 1, barcode 3, miracidia 1; (d) index 1, barcode 4, miracidia 2; (e) index 2, barcode 1, miracidia 3; (f) index 2, barcode 2, miracidia 7; (g) index 2, barcode 3, miracidia 8; (h) index 2, barcode 4, miracidia 4. Note that (a) is the same graph as shown in Fig 4. (TIF) [file pntd.0005292.s004.tif]
