# Supplementary material for: Whole Genome Amplification and Reduced-Representation Genome Sequencing of Schistosoma japonicum Miracidia
Source: PLoS Negl Trop Dis. 2017 Jan 20;11(1):e0005292. doi: 10.1371/journal.pntd.0005292 (PMC5287463; doi:10.1371/journal.pntd.0005292)
Supplement: S1 Table — Sequences of olionucleotides used as adaptors and PCR primers. (PDF) [file pntd.0005292.s005.pdf]

S1 Table. ddRADSeq adaptor and primer sequences

Type	ID	Sequence (5'-3')
Adaptor	Barcode1	ACACTCTTTCCCTACACGACGCTCTTCCGATCTNNNNNNNNTCGATTGCA<br>ATCGANNNNNNNNAGATCGGAAGAGCGTCGTGTAGGGAAAGAGTGT
Adaptor	Barcode2	ACACTCTTTCCCTACACGACGCTCTTCCGATCTNNNNNNNNCGATCTGCA<br>GATCGNNNNNNNNAGATCGGAAGAGCGTCGTGTAGGGAAAGAGTGT
Adaptor	Barcode3	ACACTCTTTCCCTACACGACGCTCTTCCGATCTNNNNNNNNAACCATGCA<br>TGGTTNNNNNNNNAGATCGGAAGAGCGTCGTGTAGGGAAAGAGTGT
Adaptor	Barcode4	ACACTCTTTCCCTACACGACGCTCTTCCGATCTNNNNNNNNGCATGTGCA<br>CATGCNNNNNNNNAGATCGGAAGAGCGTCGTGTAGGGAAAGAGTGT
Adaptor	Universal	GTGACTGGAGTTCAGACGTGTGCTCTTCCGATCTNNNNNNNN<br>GATCNNNNNNNNAGATCGGAAGAGCGAGAACAA
Primer	Index1	CAAGCAGAAGACGGCATACGAGATCGTGATGTGACTGGAGTTCAGACGTGTGC
Primer	Index2	CAAGCAGAAGACGGCATACGAGATACATCGGTGACTGGAGTTCAGACGTGTGC
Primer	Universal	AATGATACGGCGACCACCGAGATCTACACTCTTTCCCTACACGACG
