# Supplementary material for: Whole Genome Amplification and Reduced-Representation Genome Sequencing of Schistosoma japonicum Miracidia
Source: PLoS Negl Trop Dis. 2017 Jan 20;11(1):e0005292. doi: 10.1371/journal.pntd.0005292 (PMC5287463; doi:10.1371/journal.pntd.0005292)
Supplement: S2 Table — Index barcode combinations correspond to individual miracidia as follows: index 1, barcode 1, miracidia 5; index 1, barcode 2, miracidia 6; index 1, barcode 3, miracidia 1; index 1, barcode 4, miracidia 2; index 2, barcode 1, miracidia 3; index 2, barcode 2, miracidia 7; index 2, barcode 3, miracidia 8; index 2, barcode 4, miracidia 4. (PDF) [file pntd.0005292.s006.pdf]

S2 Table. Number of fragments sequenced between 300 and 600 bp recovered in each miracidium sample

	Index1				Index2				Both
Depth	Barcode1	Barcode2	Barcode3	Barcode4	Barcode1	Barcode2	Barcode3	Barcode4	1 thru 4
10x	12,602	13,343	12,445	13,125	12,878	9,928	12,702	13,405	15,404
20x	10,899	11,930	10,572	11,726	11,212	7,379	11,027	11,993	15,155

Index barcode combinations correspond to individual miracidia as follows: index 1, barcode 1, miracidia 5; index 1, barcode 2, miracidia 6; index 1, barcode 3, miracidia 1; index 1, barcode 4, miracidia 2; index 2, barcode 1, miracidia 3; index 2, barcode 2, miracidia 7; index 2, barcode 3, miracidia 8; index 2, barcode 4, miracidia 4.
