# Supplementary material for: Whole Genome Amplification and Reduced-Representation Genome Sequencing of Schistosoma japonicum Miracidia
Source: PLoS Negl Trop Dis. 2017 Jan 20;11(1):e0005292. doi: 10.1371/journal.pntd.0005292 (PMC5287463; doi:10.1371/journal.pntd.0005292)
Supplement: S3 Table — Index barcode combinations correspond to individual miracidia as follows: index 1, barcode 1, miracidia 5; index 1, barcode 2, miracidia 6; index 1, barcode 3, miracidia 1; index 1, barcode 4, miracidia 2; index 2, barcode 1, miracidia 3; index 2, barcode 2, miracidia 7; index 2, barcode 3, miracidia 8; index 2, barcode 4, miracidia 4. (PDF) [file pntd.0005292.s007.pdf]

S3 Table. Number of fragments under 600 bp sequenced in each miracidium sample

	Index1				Index2				Both
Depth	Barcode1	Barcode2	Barcode3	Barcode4	Barcode1	Barcode2	Barcode3	Barcode4	All
10x	32,804	35,550	33,039	35,070	34,074	25,045	34,116	35,728	43,156
20x	26,794	31,289	27,159	30,986	39,046	17,659	29,266	31,674	41,825

Index barcode combinations correspond to individual miracidia as follows: index 1, barcode 1, miracidia 5; index 1, barcode 2, miracidia 6; index 1, barcode 3, miracidia 1; index 1, barcode 4, miracidia 2; index 2, barcode 1, miracidia 3; index 2, barcode 2, miracidia 7; index 2, barcode 3, miracidia 8; index 2, barcode 4, miracidia 4.
