# Supplementary material for: Whole Genome Amplification and Reduced-Representation Genome Sequencing of Schistosoma japonicum Miracidia
Source: PLoS Negl Trop Dis. 2017 Jan 20;11(1):e0005292. doi: 10.1371/journal.pntd.0005292 (PMC5287463; doi:10.1371/journal.pntd.0005292)
Supplement: S5 Table — Pairwise comparison of similarity between 8 miracidia samples at 67,525 bi-allelic variants. The mean similarity is depicted as the top number in each cell, with the mean ± 2 standard deviations shown as the bottom row in each cell. Shading within cells corresponds to the degree of similarity for the two miracidia being compared, with darkers shades of gray indicating more similarity. (PDF) [file pntd.0005292.s009.pdf]

S5 Table. Genotype sharing among eight miracidia samples

			Village A				Village B			
			Individual 1			Individual 2	Individual 3			
			Miracidium 1	Miracidium 2	Miracidium 3	Miracidium 4	Miracidium 5	Miracidium 6	Miracidium 7	Miracidium 8
Village A	Individual 1	Miracidium 1	1<br>1 1	0.870<br>0.868 0.871	0.904<br>0.903 0.906	0.812<br>0.810 0.815	0.788<br>0.786 0.790	0.791<br>0.789 0.793	0.788<br>0.786 0.791	0.770<br>0.768 0.773
		Miracidium 2	0.870<br>0.868 0.871	1<br>1 1	0.869<br>0.867 0.871	0.786<br>0.784 0.788	0.764<br>0.761 0.766	0.764<br>0.762 0.767	0.766<br>0.764 0.769	0.788<br>0.786 0.790
		Miracidium 3	0.904<br>0.903 0.906	0.869<br>0.867 0.871	1<br>1 1	0.812<br>0.810 0.814	0.794<br>0.791 0.796	0.790<br>0.787 0.792	0.791<br>0.789 0.793	0.767<br>0.765 0.769
	Individual 2	Miracidium 4	0.812<br>0.810 0.815	0.786<br>0.784 0.788	0.812<br>0.810 0.814	1<br>1 1	0.784<br>0.782 0.787	0.787<br>0.784 0.789	0.787<br>0.784 0.789	0.751<br>0.749 0.754
Village B	Individual 3	Miracidium 5	0.788<br>0.786 0.790	0.764<br>0.761 0.766	0.794<br>0.791 0.796	0.784<br>0.782 0.787	1<br>1 1	0.806<br>0.804 0.809	0.829<br>0.827 0.831	0.809<br>0.807 0.811
		Miracidium 6	0.791<br>0.789 0.793	0.764<br>0.762 0.767	0.790<br>0.787 0.792	0.787<br>0.784 0.789	0.806<br>0.804 0.809	1<br>1 1	0.825<br>0.823 0.827	0.773<br>0.771 0.775
		Miracidium 7	0.788<br>0.786 0.791	0.766<br>0.764 0.769	0.791<br>0.789 0.793	0.787<br>0.784 0.789	0.829<br>0.827 0.831	0.825<br>0.823 0.827	1<br>1 1	0.806<br>0.804 0.808
		Miracidium 8	0.770<br>0.768 0.773	0.788<br>0.786 0.790	0.767<br>0.765 0.769	0.751<br>0.749 0.754	0.809<br>0.807 0.811	0.773<br>0.771 0.775	0.806<br>0.804 0.808	1<br>1 1

Pairwise comparison of similarity between 8 miracidia samples at 67,525 bi-allelic variants. The mean similarity is depicted as the top number in each cell, with the mean ± 2 standard deviations shown as the bottom row in each cell. Shading within cells corresponds to the degree of similarity for the two miracidia being compared, with darkers shades of gray indicating more similarity.
